# Gamma ray detection performance of newly developed MAPD-3NM-II photosensor with LaBr_3_(Ce) crystal

**DOI:** 10.1038/s41598-022-20006-z

**Published:** 2022-09-23

**Authors:** M. Holik, F. Ahmadov, A. Sadigov, G. Ahmadov, D. Berikov, F. Mamedov, J. Naghiyev, I. Stekl, Z. Sadygov, A. Mammadli, A. Garibli, O. Urban, J. Zich

**Affiliations:** 1 Faculty of Electrical Engineering, University of West Bohemia, Univerzitni 2795/26, 301 00 Pilsen, Czech Republic; 2 Institute of Experimental and Applied Physics, Czech Technical University in Prague, Husova 240/5, 110 00 Prague, Czech Republic; 3 Institute of Radiation Problems-ANAS, B. Vahabzade Str. 9, AZ1143 Baku, Azerbaijan; 4 National Nuclear Research Center Under the MDDT, Baku Shamakhy HW 20 km, Gobu Sett. of Absheron Dist, AZ0100 Baku, Azerbaijan; 5 The Institute of Nuclear Physics, Ibragimova 1, 050032 Almaty, Kazakhstan

**Keywords:** Electronics, photonics and device physics, Photonic devices

## Abstract

This paper presents the gamma-ray detection performance of the newly developed MAPD-3NM-II type SiPM sensor array (4 $\times$ 4) with $\hbox {LaBr}_3$(Ce) scintillator. The gamma-ray spectra of various sources have been measured in the energy range from 26 keV up to 1332 keV. The newly developed array based on MAPD-3NM-II sensors proved $\sim$ 22% enhancement in energy resolution in comparison to the former MAPD-3NM-I based array. The energy resolution of 662 keV gamma-rays measured by MAPD-3NM-II was 3.3% while clearly surpassing 4.25% resolution of MAPD-3NM-I predecessor. The enhancement is related to the high PDE of the new MAPD-3NM-II. Obtained results show that the new MAPD-3NM-II demonstrated good energy resolution and linearity in the studied energy region. The energy resolution of the new detector developed based on MAPD-3NM-II was better than all previous products of MAPD.

## Introduction

Recently, unmanned aerial vehicle (UAV) technology has been used widely in different fields from the army to security^1–8^. The use of drones in these areas makes it possible to reduce the risk to human life, to control the situation very quickly and accurately^1^. Drones are now commonly being used for environmental radiation monitoring and radiological mapping following a nuclear or radiological emergency, such as at Fukushima in Japan and the Chernobyl nuclear power plant in Ukraine^4–8^. The main requirements on radiation monitoring devices used in drone technology are low power consumption, light mass, humidity resistance, resistance to vibration and shock, and so on^8^. Conventional scintillation detectors based on vacuum photomultiplier tubes (PMT) do not meet these specific operational conditions. Therefore, scintillation detectors based on new photosensors are quite promising candidates that meet the requirements much better, and focusing on their further development is in high demand^9–22^. Many research teams focus on the development of scintillation detectors based on micro pixel avalanche photodiodes (MAPD) or silicon avalanche photomultipliers (SiPM)^15–26^. The parameters of latest SiPM generations allows them to become applicable as effective sensor element replacements of PMT in scintillation detectors. There are many works demonstrating the gamma-ray detection performance of scintillation detectors based on SiPM^23–27^. The main requirements for SiPM in this application are high pixel density and photon detection efficiency (PDE) which play a key role in the linearity and energy resolution of the entire detector^17,21,23–26^.

In this work, we demonstrate the gamma-ray detection performance of the new MAPD-3NM-II type SiPM sensor array assembled with $\hbox {LaBr}_3$(Ce) scintillators.

## Experimental setup

**Figure 1:**
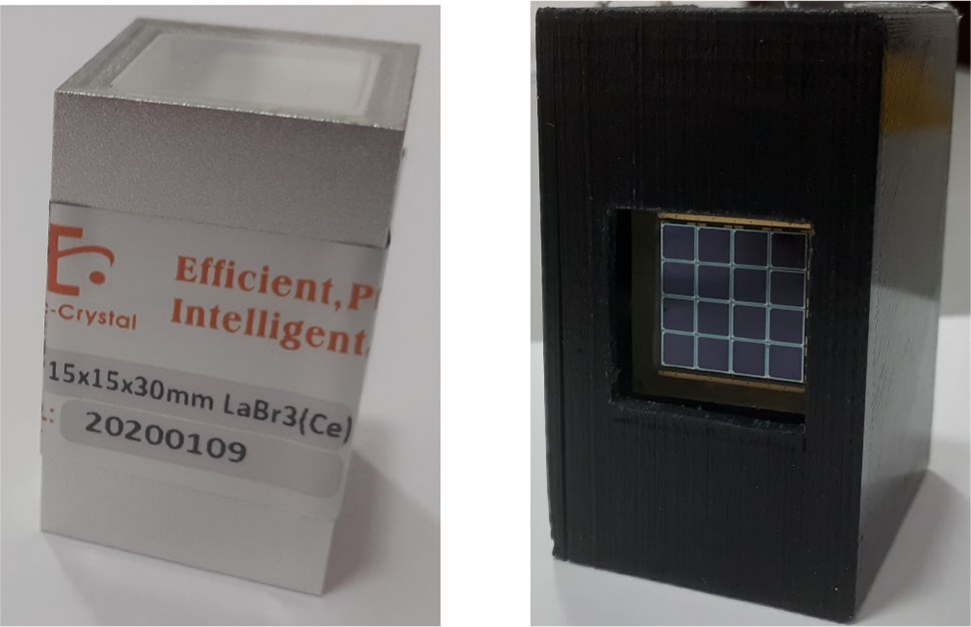
Left—photo of the $\hbox {LaBr}_3$(Ce) scintillator (15 $\times$ 15 $\times$ $30\,\hbox {mm}^3$), right—photo of MAPD-3NM-II type SiPM array embedded in the protective casing.

The cubic $\hbox {LaBr}_3$(Ce) crystal (15 $\times$ 15 $\times$ 30 $\hbox {mm}^3$) used in measurements was produced by Epic Crystal^28^. $\hbox {LaBr}_3$(Ce) crystal is hermetically sealed in a thin aluminum package with a 3 mm light guide on one face (Fig. 1 left). The scintillator was mounted on the MAPD-3NM-II type SiPM array with optical grease.

Parameters of the used scintillator are given in Table 1.

**Table 1:** Parameters of the $\hbox {LaBr}_3$(Ce) scintillator^28^.

Density (g/cm^3^)	5.10
Decay time (ns)	20
Light output (Photons/MeV)	60.000
Wavelength of emission peak (nm)	380
Energy resolution (662 keV)	< 4.5%
Refractive index	2.10
Hygroscopic	Yes

The MAPD-3NM-II is used as the photon readout of the $\hbox {LaBr}_3$(Ce) scintillator. Due to the large size of $\hbox {LaBr}_3$:Ce scintillator, 16 SiPMs of the MAPD-3NM-II type are combined in parallel to obtain a large sensitive area. MAPD-3NM-II with buried pixel structure manufactured together with Zecotek Company in 2020 (Fig. 1 right)^23,29^. The array is made up of sixteen 3.7 $\times$ 3.7 $\hbox {mm}^2$ MAPD elements and each element consists of 61000 pixels. A positive voltage was applied to the cathode, and the common anode was used as an output. The gap between two MAPD elements is 0.1 mm, as designed in the PCB layout. The total area covered by the 16 MAPDs is 17 $\times$ 17 $\hbox {mm}^2$. The technical specifications and parameters of the MAPD-3NM-II array are listed in Table 2.

**Table 2:** Technical specifications and parameters of the MAPD array.

Manufacture	Zecotek photonics
Type	MAPD-3NM-II
Active area	17 × 17 mm^2^
Channels	16 (4 × 4)
Pixel pitch/pixel (diameter)	15 μm/ 12 μm
Total pixels	974 728 (6 1000 pixels/channel)
Fill factor	76%
Gain	~ 2.5 × 10^5^
Spectral response	300–900 nm (max at 450 nm)
Operation voltage range	54–56 V
Breakdown voltage	51.6 V
Capacitance/channel	2 480/155 pF

## Results and discussion

The operation and breakdown voltage of MAPD array is defined from the relative derivative of the I–V curve. The reverse current–voltage (I–V) characteristic is performed using a Keithley 6487 Picoammeter/Voltage Source. The breakdown voltage ($\hbox {U}_{{br}}$) was 51.6 V and the full working voltage range of the MAPD-3NM-II array was 54–56 V.

The total capacitance of the array was $\sim$ 2.5 nF. Compared to the previous MAPD-3NM-I element, the following parameters of the new MAPD-3NM-II was improved thanks to optimization of the structure: the operating voltage decreased by 24%, the dark current decreased more than 2 times at the same gain, the capacitance decreased by 23%, the gain increased 2 times, and the PDE increased more than 30%^23^.

This experiment was performed with various radio-isotope standard sources. The gamma-ray detection performance of the detector is tested with $^{241}\hbox {Am}$, $^{133}\hbox {Ba}$, $^{137}\hbox {Cs}$, and $^{60}\hbox {Co}$ point radioactive sources. The parameters of radioactive sources are shown in Table 3. The radioactive sources were placed on the top of the $\hbox {LaBr}_3$(Ce) scintillator inside the dark box.

**Table 3:** The parameters of radioactive sources.

Radionuclide	Activity [kBq]	X-ray [keV]	Gamma-ray energy [keV]
$^{241}\hbox {Am}$	96.72	16.6	26.34, 33.20, 43.42, 59.54, 98.97, 102.98
$^{133}\hbox {Ba}$	90	30.62, 30.97, 34.9, 34.5	53.16,79.62, 80.99, 160.61, 223.24, 276.40, 302.85, 356.01, 383.85
$^{137}\hbox {Cs}$	96.05	31.81, 32.19	661.66
$^{60}\hbox {Co}$	80.24		1173.24, 1332.51

Gamma-ray measurements were carried out in the SPECTRIG MAPD module, where the temperature was $23.5\, ^{\circ }\hbox {C}$. The detailed information about the SPECTRIG MAPD module was presented in^22^. During measurement with SPECTRIG MAPD following parameters were selected: gate width – 227 nsec, variable gain – 1 dB (for M MAPD-3NM-II) and 10 dB (for MAPD-3NM-I), threshold – 6 mV, applied voltage – 54.5 V, 55.8 V (for MAPD-3NM-II) and 76.5 V (for MAPD-3NM-I). High-energy gamma rays (e.g. E >2 MeV) did not appear in the spectrum due to saturation of the ADC output in the case of measurements with MAPD-3NM-II at different voltages. A measurement time of 5 minutes for each cycle was selected.

**Figure 2:**
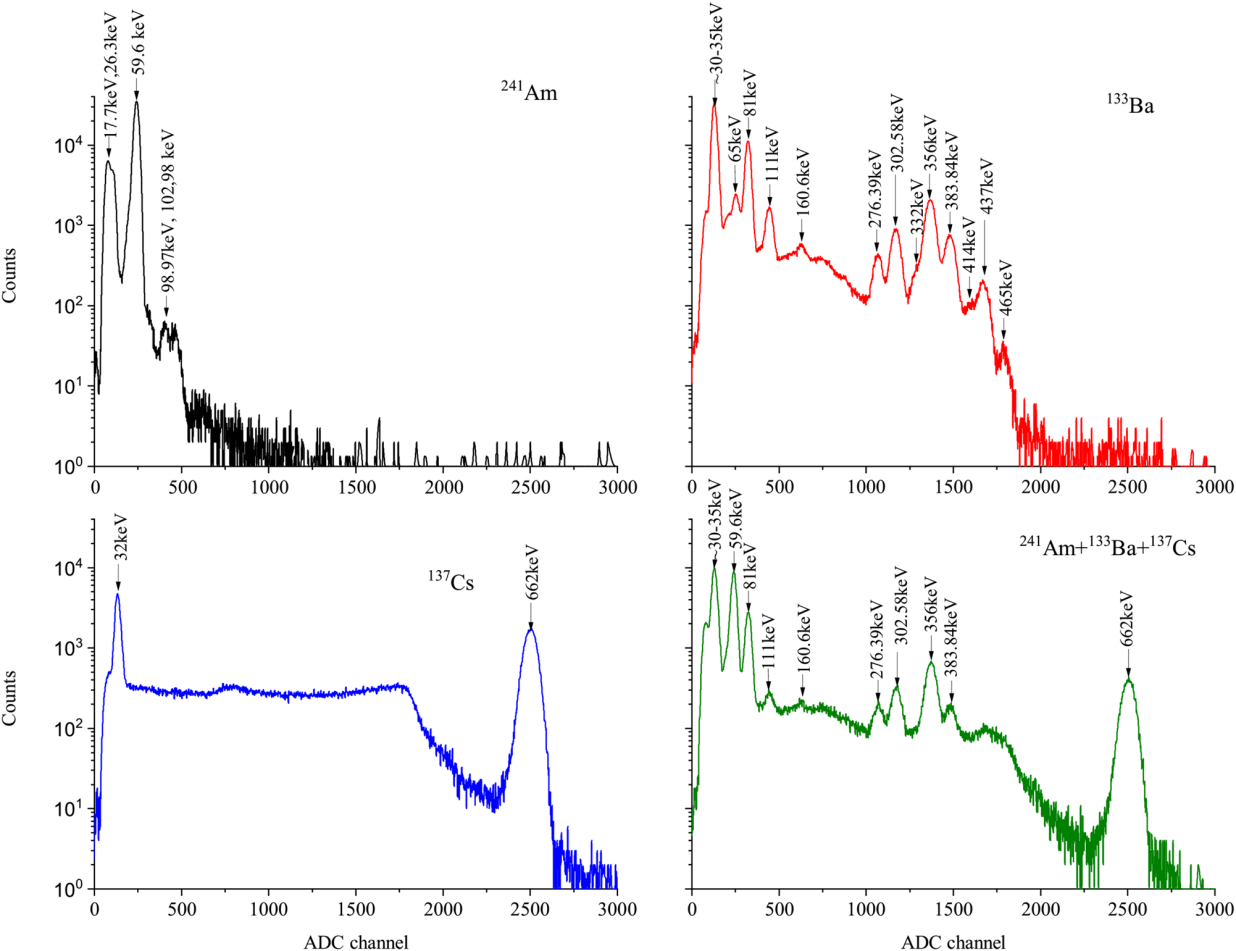
Energy spectra of mixed radioisotope sources containing ($^{241}\hbox {Am}$, $^{133}\hbox {Ba}$ and $^{137}\hbox {Cs}$) at an applied voltage of 55.8 V for $\hbox {LaBr}_3$(Ce) + MAPD-3NM-II.

Figure 2 shows the spectrum of the mixed radioisotope sources containing ($^{241}\hbox {Am}$, $^{133}\hbox {Ba}$ and $^{137}\hbox {Cs}$).

The high photon detection efficiency of MAPD-3NM-II and the high light output of the $\hbox {LaBr}_3$ scintillator made it possible to obtain a good resolution. All this made it possible to separate the photopeak of the gamma-rays. It is obvious that the newly developed MAPD detector allows detecting most of the gamma lines emitted from $^{133}\hbox {Ba}$ radioisotope. This kind of detector allowed to separate close gamma lines, e.g. 59.6 keV ($^{241}\hbox {Am}$) from 81 keV (difference $\sim$ 21 keV), 276.3 keV from 302.6 keV (difference $\sim$ 26 keV) and 356 keV from 383.8 keV (difference $\sim$ 28 keV). The energy resolution was evaluated by fitting the photopeak of the $^{137}\hbox {Cs}$ energy spectrum with a Gaussian function. The energy resolution of 662 keV gamma rays was 3.3%.

In the case of measurements of $^{60}\hbox {Co}$ with MAPD-3NM-II at 55.8 V voltage, the output of the ADC of SPECTRIG MAPD module was saturated (even when a variable gain was set 1 dB, i.e. minimal) that is why high-energy gamma-rays (e.g. E > 1 MeV) was not seen at this voltage. This disadvantage was avoided by decreasing the operation voltage to 54.5 V. However, as a side-effect of this approach, the energy resolution of the detector gets reduced but detection of high-energy gamma-rays (e.g. E > 1 MeV) is possible.

**Figure 3:**
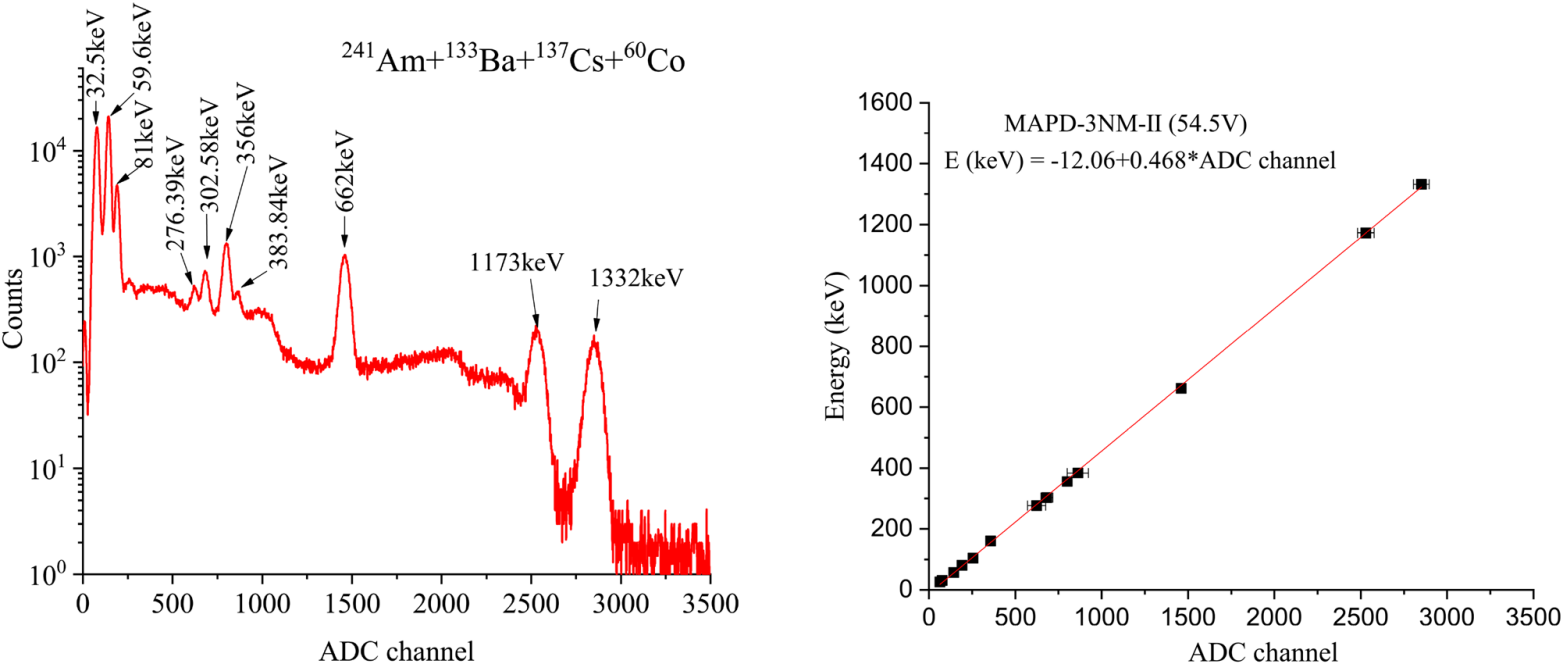
Energy spectra of the $^{241}\hbox {Am}$ + $^{133}\hbox {Ba}$ + $^{137}\hbox {Cs}$ + $^{60}\hbox {Co}$ (left) and pulse height (right) of gamma-ray signals as a function of energy at an applied voltage of 54.5 V for $\hbox {LaBr}_3$(Ce) + MAPD-3NM-II. If no error bar is shown, the error is contained within the point.

A spectrum of mixed radioactive sources containing $^{241}\hbox {Am}$ + $^{133}\hbox {Ba}$ + $^{137}\hbox {Cs}$ + $^{60}\hbox {Co}$ is depicted in Fig. 3 (left).

**Figure 4:**
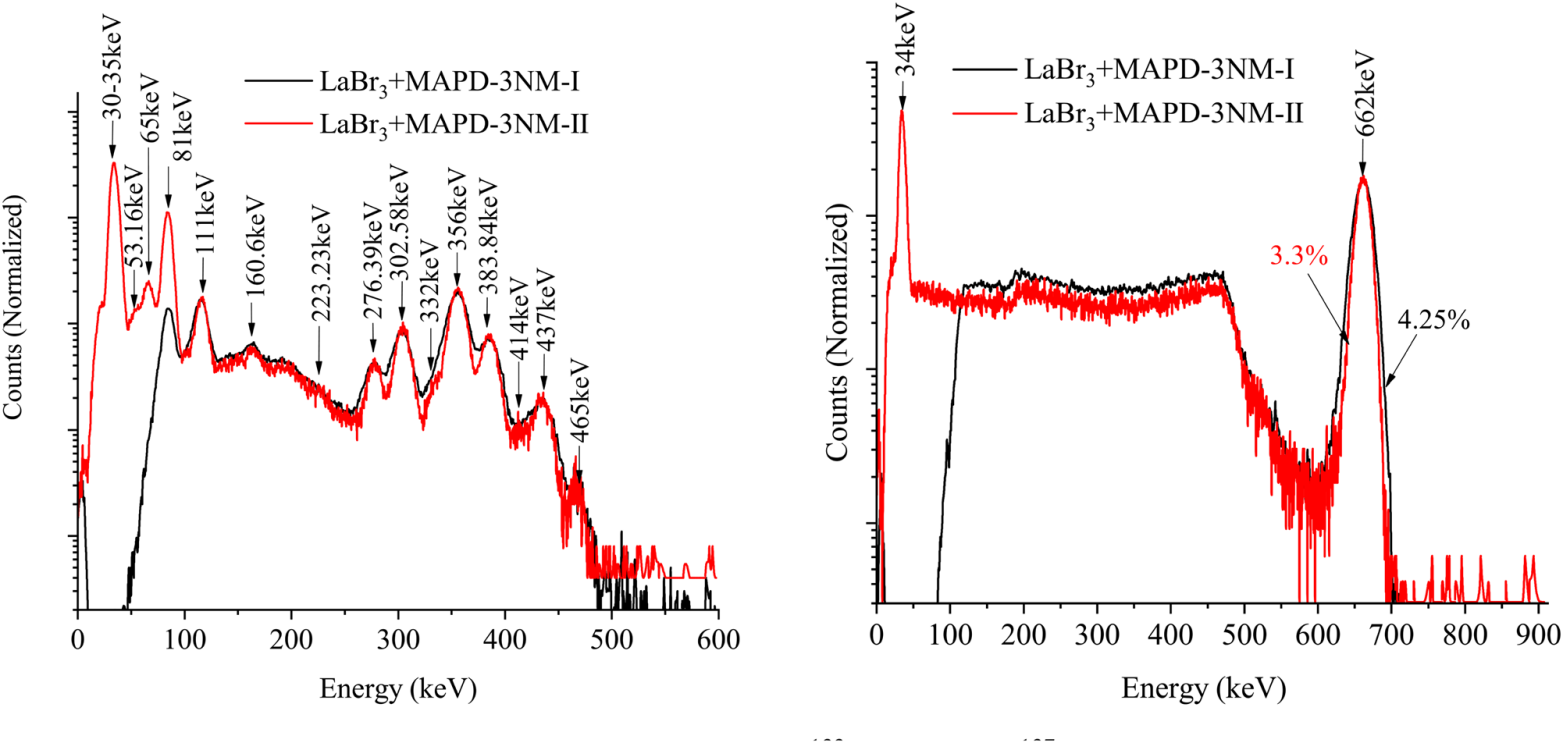
Comparison of normalized pulse height spectrum for $^{133}\hbox {Ba}$ (left) and $^{137}\hbox {Cs}$ (right) with $\hbox {LaBr}_3$(Ce) and MAPD-3NM array (I—black and II—red).

Figure 4 shows a comparison of pulse height spectrum for $^{133}\hbox {Ba}$ and $^{137}\hbox {Cs}$ with $\hbox {LaBr}_3$(Ce) and MAPD-3NM array (I – black (75 V) and II – red (55.8 V)). We can see all gamma lines of $^{133}\hbox {Ba}$ radioisotope in the spectrum. All photopeaks in the energy range 30 - 456 keV are well separated (Fig. 4 left). The corresponding resolution of the detector varies from 7 to 29% depending on the energy of gamma-ray and PDE of the MAPD - 3NM array.

The energy resolutions of 662 keV gamma-rays measured by MAPD-3NM-I and 3NM-II array were 4.25% and 3.3% (Fig. 4 right). MAPD-3NM-II demonstrated $\sim$ 22% better energy resolution compared to the MAPD-3NM-I^21^. This improvement was due to the high PDE (33%) of the MAPD-3NM-II^23^.

**Figure 5:**
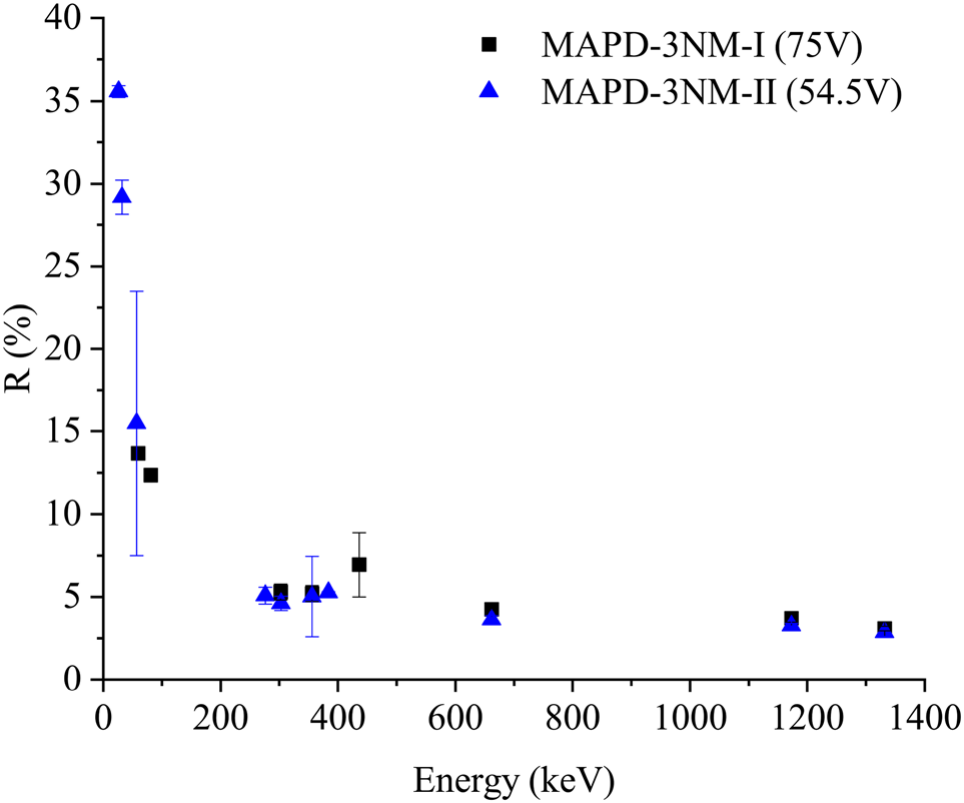
The energy resolution of gamma-ray signals as a function of energy. If no error bar is shown, the error is contained within the point.

Variation of energy resolution for gamma-ray energies of 26, 32, 56.9, 276.39, 302.85, 356, 383.84, 662, 1173, and 1332 keV were depicted in Fig. 5. The energy resolution of the detectors varied in the range of 3 - 13.6% for MAPD-3 NM-I array (59.6 - 1332 keV, at 75 V) and 2.8 - 35% MAPD-3NM-II array (30 - 1332 keV, at 54.5 V).

**Figure 6:**
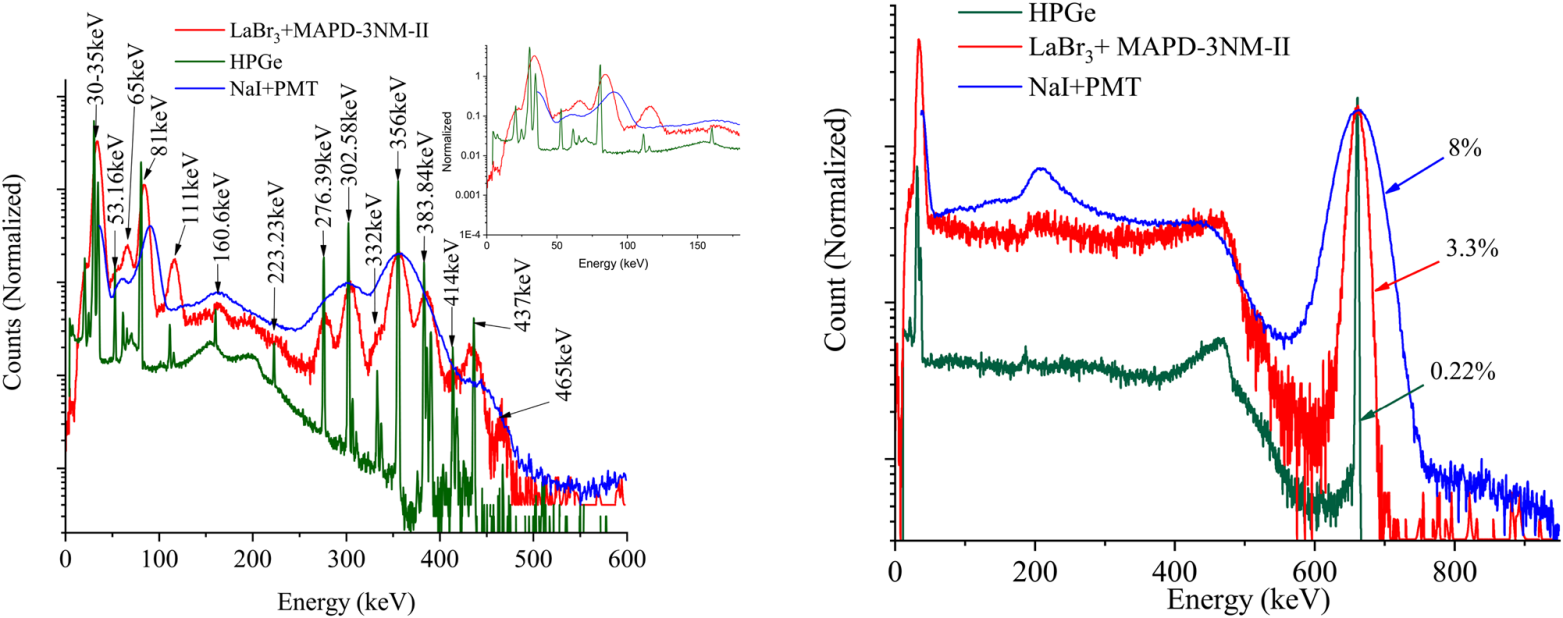
Comparison of normalized pulse height spectrum for $^{133}\hbox {Ba}$ (left) and $^{137}\hbox {Cs}$ (right) with HPGe, NaI(Tl) + PMT and $\hbox {LaBr}_3$(Ce) + MAPD-3NM-II (55.8 V) detectors.

In Fig. 6 shows a comparison of the energy spectra for $^{133}\hbox {Ba}$ and $^{137}\hbox {Cs}$ sources acquired with three different gamma detectors: commercial coaxial HPGe detector and identiFINDER-2 device (green and blue spectra, respectively) and the new developed $\hbox {LaBr}_3$(Ce) + MAPD-3NM-II (red spectrum). The HPGe model GX3020 was manufactured by CANBERRA, while the identiFINDER-2, composed by a NaI(Tl) crystal (size Ø31 mm $\times$ 51 mm) coupled to a PMT, was manufactured by ICx Technologies, Inc. The HPGe detector is used to clearly identified the position of gamma-rays emitted by $^{133}\hbox {Ba}$ (Table 3). We note that there are several gamma lines associated with sum peaks (81 keV + 30 keV = 111 keV, 356 keV + 81 keV = 437 keV, 384 keV + 81 keV = 465 keV). When NaI(Tl) + PMT and $\hbox {LaBr}_3$(Ce) + MAPD-3NM-II detectors were used, the gamma-ray spectra of radioactive sources were taken without lead shielding assemblies, therefore the natural background affected the spectra. It can be seen how the $\hbox {LaBr}_3$(Ce) + MAPD-3NM-II is able to resolve virtually all the gamma-rays emitted by $^{133}\hbox {Ba}$. The non-linearity of $\hbox {LaBr}_3$(Ce) scintillator is visible in the lower energy region of $^{133}\hbox {Ba}$ spectrum. In Fig. 6 is shown the spectrum of $^{133}\hbox {Ba}$ with NaI(Tl) + PMT, too. The poor resolution of the NaI(Tl) + PMT detector does not allow separation of the gamma-rays emitted by $^{133}\hbox {Ba}$. As for the energy spectra of $^{137}\hbox {Cs}$ ( Fig. 6 right part), the Compton edge and backscatter peak of gamma-rays emitted for 662 keV gamma-ray is clearly visible. The energy resolutions of 662 keV gamma-rays measured by NaI(Tl) + PMT and $\hbox {LaBr}_3$(Ce) + MAPD-3NM were 8% and 3.3%, respectively. As is expected due to the improved relative energy resolution of the $\hbox {LaBr}_3$(Ce) than NaI(Tl) crystal, the new $\hbox {LaBr}_3$(Ce) + MAPD-3NM shows a $\sim$ 2.4 times better energy resolution than the NaI(Tl) + PMT. The parameters of the detectors used for comparison are given in Table 4.

**Table 4:** The parameters of tested detectors.

Type	HPGe (Coaxial Detector model GX3020)	NaI(Tl) + PMT (identiFINDER-2)	$\hbox {LaBr}_3$(Ce) + MAPD-3NM-II type SiPM
Operation voltage (V)	+ 3000	600–1000	56
Operation temperature (°C)	− 195	+ 25	+ 25
Energy resolution for 662 keV (%)	0.22	8	3.3
Size	Ø60 mm × 50 mm	Ø31 mm × 51 mm	15 mm × 15 mm × 30 mm

**Figure 7:**
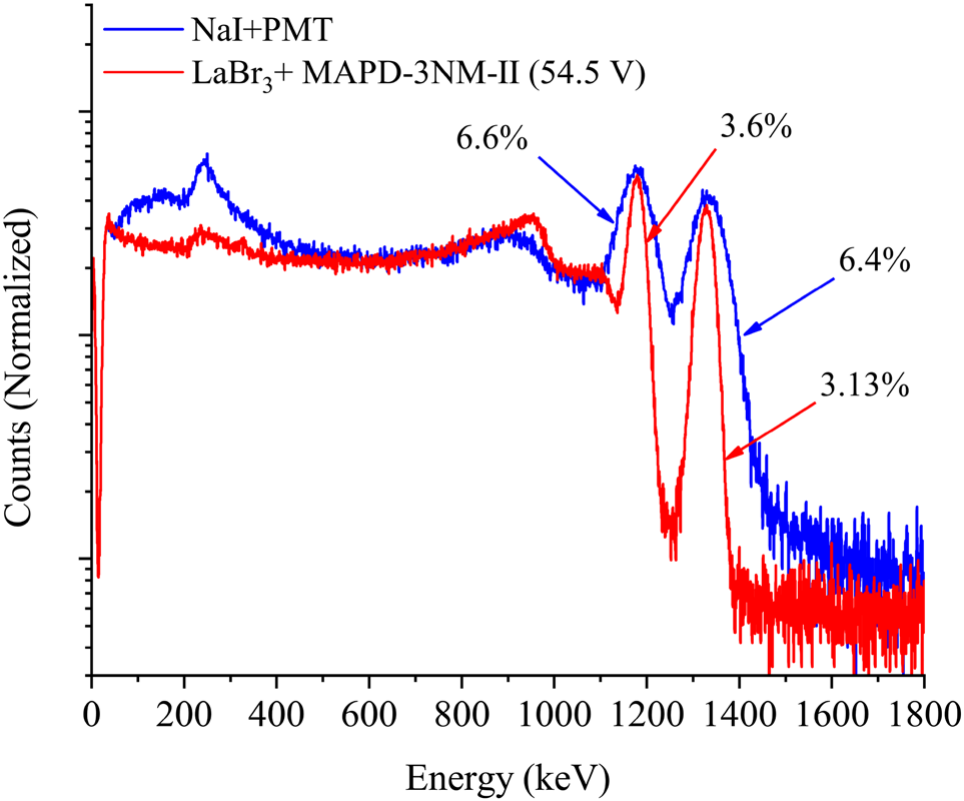
Comparison of normalized pulse height spectrum for $^{60}$Co with NaI(Tl) + PMT and $\hbox {LaBr}_3$(Ce) + MAPD-3NM-II (54.5 V) detectors.

In the case of measurements of $^{60}$Co with MAPD-3NM-II has applied 54.5 V voltage, due to avoid the saturation of the ADC output of the SPECTRIG MAPD module. Reducing the voltage will reduce the photon detection efficiency of the MAPD-3NM-II and degrade the energy resolution of the detectors. $\hbox {LaBr}_3$(Ce) + MAPD-3NM-II (54.5 V) demonstrated $\sim$ 1.8 times better energy (for 1173.2 keV and 1332.5 keV) resolution compared to the NaI(Tl) + PMT (Fig. 7).

Obtained results proved that the newly designed MAPD-3NM-II array demonstrated good energy resolution and linearity in the wide energy region. The maximum detectable energy was 1332 keV and the number of fired pixels was about 21000 in this case ($\sim$ 40 times smaller than the total number of pixels of the array). The number of fired pixels was calculated by taking into account PDE (30% for 380 nm) and geometrical factor of MAPD-3NM array (76% for 4 $\times$ 4 array). It means that MAPD-3NM-II with $\hbox {LaBr}_3$(Ce) scintillators can be successfully used to detect tens MeV gamma-rays too. The maximum value of the recorded energy was limited due to the saturation of the ADC output in this work. In the future, it is planned to develop the following parameters of the SPECTRIG MAPD module: extending the dynamic range of the ADC up to 2 V, adding variable attenuator to the output of the MAPD, and adding a temperature compensation circuit to keep the MAPD operating with a constant gain.

## Conclusion

The gamma-ray detection performance of the new MAPD-3NM-II array with $\hbox {LaBr}_3$ scintillator has been studied in the energy range from 26 keV up to 1330 keV. The $\hbox {LaBr}_3$:Ce scintillator coupled to MAPD-3NM-II achieves an energy resolution of 3.3% at 662 keV and 4.5% for MAPD-3NM-I. The better energy resolution was related to the high PDE of the new MAPD-3NM-II photodiodes. The $\hbox {LaBr}_3$ + MAPD-3NM-II unit delivers 3.3% more energy resolution at 662 keV than the Nal(Tl) + PMT unit, which only gives 8%. So, in other words, $\hbox {LaBr}_3$ provides better resolution performance over NaI(Tl) systems by approximately a factor of 2.4. Note that neither the NaI(Tl) detectors nor the lanthanum bromide detectors can approach the resolution of the HPGe detector due to its low band gap of 0.67 eV.

Obtained results showed that the new MAPD-3NM-II demonstrated good energy resolution and linearity in the studied energy region. The energy resolution of the new detector developed based on MAPD-3NM-II was better than all previous products of MAPD. For applications such as nuclear physics, public security, industry, space application and environmental radiation monitoring, $\hbox {LaBr}_3$ + MAPD-3NM-II unit ability to detect gamma-rays with high sensitivity is extremely important. These benefits make MAPD-3NM-II an alternative photodiode to existing instruments. In addition, MAPD-3NM-II based scintillation detectors can be considered to be more benign, in the sense that they can operate at room temperature, do not require such high voltages, increasing simplicity and a marked reduction in mass. Very small sensitive regions (4 $\mu$m) make these photodiodes less susceptible to radiation damage. All these advantages of the new MAPD-3NM-II makes them optimal for radiation monitoring devices that can be used in drone technology.

## Data Availability

The datasets obtained during the experiments, used and analyzed during this study are included in this published article and its supplementary information files. The datasets are also available from the corresponding author upon reasonable request.
